# A model for extending antiretroviral care beyond the rural health centre

**DOI:** 10.1186/1758-2652-12-22

**Published:** 2009-09-29

**Authors:** Kara K Wools-Kaloustian, John E Sidle, Henry M Selke, Rajesh Vedanthan, Emmanuel K Kemboi, Lillian J Boit, Viola T Jebet, Aaron E Carroll, William M Tierney, Sylvester Kimaiyo

**Affiliations:** 1 Department of Medicine, Indiana University School of Medicine, Indianapolis, USA; 2 Zena and Michael A Wiener Cardiovascular Institute, Mount Sinai Medical Center, New York, USA; 3 United States Agency for International Development - Academic Model for Providing Access to Healthcare (USAID-AMPATH) Partnership, Edoret, Kenya; 4 Department of Pediatrics, Indiana University School of Medicine, Indianapolis, USA; 5 Regenstrief Institute, Indianapolis, USA; 6 Department of Medicine, Moi University Faculty of Health Sciences, Eldoret, Kenya

## Abstract

**Background:**

A major obstacle facing many lower-income countries in establishing and maintaining HIV treatment programmes is the scarcity of trained health care providers. To address this shortage, the World Health Organization has recommend task shifting to HIV-infected peers.

**Methods:**

We designed a model of HIV care that utilizes HIV-infected patients, community care coordinators (CCCs), to care for their clinically stable peers with the assistance of preprogrammed personal digital assistants (PDAs). Rather than presenting for the standard of care, monthly clinic visits, in this model, patients were seen every three months in clinics and monthly by their CCCs in the community during the interim two months. This study was conducted in Kosirai Division, western Kenya, where eight of the 24 sub-locations (defined geographic areas) within the division were randomly assigned to the intervention with the remainder used as controls.

Prior to entering the field, CCCs underwent intensive didactic training and mentoring related to the assessment and support of HIV patients, as well as the use of PDAs. PDAs were programmed with specific questions and to issue alerts if responses fell outside of pre-established parameters. CCCs were regularly evaluated in six performance areas. An impressionistic analysis on the transcripts from the monthly group meetings that formed the basis of the continuous feedback and quality improvement programme was used to assess this model.

**Results:**

All eight of the assigned CCCs successfully passed their training and mentoring, entered the field and remained active for the two years of the study. On evaluation of the CCCs, 89% of their summary scores were documented as superior during Year 1 and 94% as superior during Year 2. Six themes emerged from the impressionistic analysis in Year 1: confidentiality and "community" disclosure; roles and responsibilities; logistics; clinical care partnership; antiretroviral adherence; and PDA issues. At the end of the trial, of those patients not lost to follow up, 64% (56 of 87) in the intervention and 52% (58 of 103) in the control group were willing to continue in the programme (p = 0.26).

**Conclusion:**

We found that an antiretroviral treatment delivery model that shifted patient monitoring and antiretroviral dispensing tasks into the community by HIV-infected patients was both acceptable and feasible.

**Trial registration:**

ClinicalTrials.gov ID NCT00371540

## Introduction

Two-thirds of the approximately 33 million HIV-infected people globally reside in the resource-constrained countries of sub-Saharan Africa, where more than 50% of the population lives in rural areas [1,2]. Though often thought of as an urban epidemic, rural HIV prevalence ranges from 5.3 to 21.9% in Eastern and Southern Africa [3]. The clinical benefits of antiretroviral treatment (ART) for individuals residing in resource-poor settings have been documented in multiple studies [4-10]. Despite this documented benefit and a concerted international effort to roll out ART, only four countries in sub-Saharan Africa (Senegal, Rwanda, Botswana and Namibia) have achieved the "3 by 5" goal of treating at least half of the persons who are living with HIV/AIDS and need treatment[11]. A major obstacle faced by many lower-income countries is establishing and maintaining HIV treatment programmes in rural areas, where trained health care providers and adequate infrastructure are scarce [12,13].

The human resources necessary for delivery of HIV care are substantial. For example, it has been estimated that in order for Moi Teaching and Referral Hospital (MTRH), the second national referral hospital in Kenya to meet the needs of all HIV-infected patients in its catchment area of 13 million people, it will need 730 physicians and/or clinical officers (mid-level practitioners equivalent to a US nurse practitioner or physician's assistant) trained in HIV care protocols [14]. Given World Health Organization (WHO) estimates of a shortfall of 817,992 health care providers (doctors, midwives and nurses) in the African region, it will be impossible to meet the existing demand for antiretroviral care if we continue to rely on the traditional physician-, clinical officer- and nurse-based model of ART delivery [15]. Therefore, to maximize access to ART in resource-poor settings, leaders in international health have advocated the decentralization of HIV care, use of existing infrastructure, and a shift from physician-centred care models to those utilizing non-physician health workers trained in simplified and standardized approaches to care [12,15-17]. However, experience with feasible models of such "task shifting" in HIV care is limited [18-21].

To address issues related to provider resources and access to HIV care in a rural setting, we designed and implemented a model of HIV care that utilizes trained HIV-infected peers (community care coordinators, or CCCs) to care for clinically stable HIV patients within their communities. This paper presents data on the development, structure and acceptability of this model. Specifically, we describe the implementation of this innovative, community-based HIV-care programme, and present the data collected as part of the continuous feedback and quality improvement programme that was integrated into this model. There is an ongoing cluster-randomized controlled trial that is being used to assess patient outcomes within this new model, the results of which will be presented in a subsequent paper.

## Methods

This study was approved by the Indiana University School of Medicine Institutional Review Board and the Moi University Institutional Research and Ethics Committee.

### Setting

**Figure 1:**
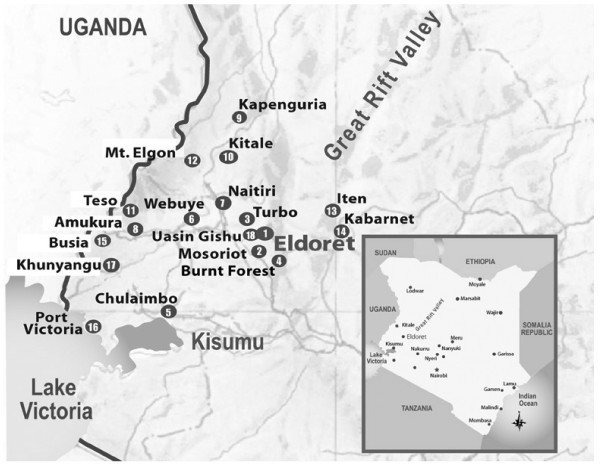
**Map of USAID-AMPATH Partnership sites**.

This study was conducted at one of the 18 primary United States Agency for International Development - Academic Model Providing Access to Healthcare (USAID-AMPATH) Partnership clinics in western Kenya (Figure 1). This HIV-care network consists of a partnership between Moi University Teaching and Referral Hospital, Moi University School of Medicine and several US-based medical schools led by Indiana University [4]. The network, headquartered in Eldoret Kenya, has been operational since November 2001 and currently cares for more than 75,000 patients, 33,000 of whom are receiving combination antiretroviral therapy (cART).

This study was conducted within the HIV clinic and the community surrounding the Mosoriot Rural Health Center (the first rural health centre to host an AMPATH clinic), located in Kosirai Division, 30 km southwest of Eldoret (Figure 1). Mosoriot serves a community of almost 40,000, with a documented HIV prevalence of 7.4% in the province [22]. As of March 2008 when this study was completed, 3,442 adult patients were in care at the Mosoriot HIV clinic, with 1,845 receiving cART. The clinic is staffed by three clinical officers for five days a week with a physician present on one day per week.

Kosirai Division is parcelled into nine smaller administrative areas, called locations, that in turn are divided into 24 sub-locations. The average sub-location is 4 km in diameter and thus can be crossed easily on foot (one to two hours). As part of our clinical trial, eight sub-locations were randomly assigned to the CCC intervention, and the remaining 16 were assigned to control status.

### Care models

#### Standard of care

At the time of this study, the majority of patients receiving cART were scheduled for monthly clinic visits, while some stable patients, demonstrating good cART adherence and living a significant distance from the Mosoriot HIV clinic, were occasionally scheduled for visits every two months. A physician attended the clinic on one day per week, reviewed difficult cases and made decisions related to opportunistic infection treatment, as well as drug substitutions for toxicity and failure that fell outside the standard AMPATH guidelines (Table 1). The physician also relieved the clinical officers of some of their routine cases.

**Table 1:** Task shifting with the CCC model (derived from WHO Task Shifting: Global Recommendations and Guidelines)

	**Standard of care**			**CCC model**			
	
	**P**	**CO**	**N**	**P**	**CO**	**N**	**CCC**
	
***Clinical monitoring***							
	
Monitor and support ART adherence	*□□□*	■■■	■■■	*□*	■	■	■■
	
Take weight			■■■			■	■■
	
Take vitals			■■■			■	■■
	
Determine functional status	■■■	■■■		*□*	■		■■
	
Request CD4 count and viral load	*□□□*	■■■		*□□□*	■■■		
	
Identify ART side effects	*□□□*	■■■		*□*	■		■■
	
Manage ART side effects	*□□□*	■■■	*□□□*		■■■		
	
Identify OI symptoms	*□□□*	■■■		*□*	■		■■
	
Manage OIs	*□□□*	■■■		*□□□*	■■■		
	
***Dispense and arrange follow-up visits***							
	
Dispense ART and drugs for OI prophylaxis			■■■			■	■■
	
Arrange follow-up visits			■■■			■	■■
	
***Manage substitutions or switch of ART***							
	
Switch to alternative first-line regimen	*□□□*	■■■		*□□□*	■■■		
	
Switch second-line regimen	*□□□*	■■■		*□□□*	■■■		
	
Choose appropriate third-line	*□□□*			*□□□*			
	
***Supervision***							
	
Clinical officers	*X*	X		X	*X*		
	
Nurses		X			X		
	
Community care coordinators					X		

X Responsible for an activity

■■■ Responsible during all visits

■■ Responsible during two-thirds of visits

■ Responsible during one-third of visits

*□□□*; *□*; *X *Physicians are responsible for all or part of the activity when present in the clinic

ART: antiretroviral treatment

OI: opportunistic infection

In collaboration with the clinical officer in charge, the physician also provided supervision, support and continuing education to the nurses and clinical officers practicing at the Mosoriot HIV clinic. For patients receiving cART, the clinical officers monitored and supported cART adherence, assessed functional status and symptoms, managed cART side effects, and diagnosed and treated opportunistic infections. The nurses were responsible for obtaining weights, vital signs, assisting in cART adherence monitoring and support, as well as dispensing ART and drugs for the prophylaxis and treatment of opportunistic infections.

#### CCC model

Under the CCC model, patients were seen every three months in the clinic and received the standard care from nurses, clinical officers and physicians, as described. During the interim two months, CCCs visited patients in their communities in locations that were mutually agreed on by the CCCs and their clients (e.g., patient's house, CCC's house or a public location). CCCs travelled through the community on foot or, rarely, with the use of public transport. CCCs received a salary for their activities, which was less than half that of a nurses and a third of a clinical officer's salary.

During a community visit, the CCCs measured the patient's temperature, weight and oxygen saturation with portable electronic devices, and performed a structured symptom review guided by a personal digital assistant (PDA). If a specific symptom or constellation of symptoms was identified, the PDA triggered a specified alert, which provided detailed instructions, such as contacting the clinic and reviewing the case with a clinical officer to discern whether the patient might require a formal evaluation in the clinic (see PDA programme description below). CCCs also dispensed the patients' monthly supply of cART and opportunistic infection (OI) prophylaxis.

### Population

The requirements for becoming a CCC included being HIV infected and within care at the Mosoriot HIV clinic, as well as being clinically stable on cART for a minimum of six months with 100% adherence to his or her regimen. In addition, the candidates had to be at least 18 years old, literate in either Kiswahili or English, fluent in Kiswahili and Kalenjin (the local language), interested in monitoring and assisting in HIV care, willing to maintain patient confidentiality, residing in or near a targeted sub-location, and willing to give consent to participate. Cumulative self-reported adherence data from clinic visits was used to determine the candidates' adherence to cART.

The HIV clinic staff assessed level of interest in monitoring and assisting in HIV care, as well as willingness to maintain confidentiality, based on previous interactions with these individuals in both the community and clinic setting, as well as during interviews at the time of recruitment. The Mosoriot clinical officers and nurses selected nine patients (five male and four female), who met these criteria, for training as CCCs. CCCs were paid a salary consistent with that of the outreach workers employed by AMPATH.

### Training and mentoring

Training of CCCs was comprised of both didactic and practical components. First, the CCC attended a one-week structured didactic training that included: an overview of antiretrovirals; performing symptom reviews; assessing adherence; providing general patient support; obtaining vitals; and using the PDA and the CCC programme. This was followed by two months of practical training at the Mosoriot HIV clinic, during which CCCs initially shadowed the clinical officers and clinic nurses through each department (clinical care, pharmacy, nutrition and social work). They subsequently performed independent assessments of stable patients and reviewed their findings with the clinical officer caring for the patient.

During the first month in the field, the CCCs visited their assigned patients and evaluated them as per protocol one to two days prior to the patients' regularly scheduled HIV clinic visits. The patients were subsequently seen at their scheduled monthly clinic appointment, where the clinical officers compared the findings of the CCCs with their own. Any important differences were discussed during CCC debriefing sessions. After this initial training period, the CCCs followed the model as outlined above.

### Personal digital assistants

PDAs were used as the platform for the electronic decision tool in this study because they are small, and thus in rural areas where technical support is unavailable, they can be easily mailed to a service provider for repair. Each CCC PDA was programmed with a series of questions directed toward the patient that included:

• New cough since last visit?

• Vomiting within the last 48 hours?

• Diarrhea within the last 48 hours?

• New headache since the last visit?

• Has the patient had any of the following occur since the last visit? (Answers: inability to walk, inability to talk, weakness on one side of the body, weakness on one side of the face)

• Over the last week, has the patient or a family member skipped a meal because of lack of food in the house?

• Has the patient reported or is there any evidence that there has been domestic violence in the household?

• Does the patient (if female) believe that she may be pregnant?

• Are there more than six pills in any of the antiretroviral bottles than there should be?

• Does the patient report significant difficulty with adherence?

**Figure 2:**
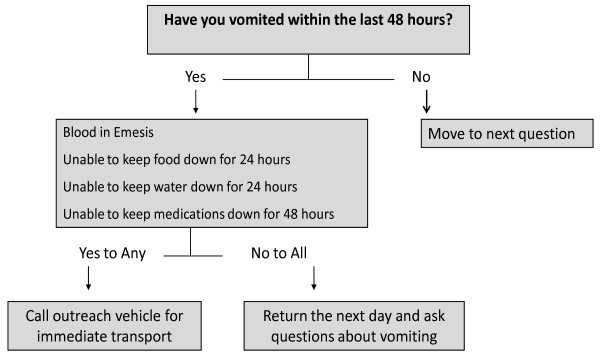
**PDA decision support algorithm for vomiting**.

An answer of "yes" to any of first five questions triggered a sub-screen that asked additional details about the symptoms. For example, with regard to the question about vomiting, the sub-screen asked for information about hematemesis, as well as the ability to keep food, water and medications down. Fields were also present for entering current temperature, weight and oxygen saturation. As noted, pre-programmed alerts were triggered if specified parameters were met. For example, with regard to the question on vomiting (Figure 2), if a "yes" response was entered into any of the sub-screen questions, an alert was displayed requiring the CCC to call the clinic via cellphone (provided to each CCC) and discuss whether that patient should be transported to the health centre. In addition, vital sign abnormalities, such as temperature ≥38.5 and oxygen saturation ≤90, triggered an alert.

### Evaluation

During the mentoring period, CCCs underwent weekly performance evaluations conducted by the in-charge clinical officer, the results of which reflected both her assessment and insights gained from the clinic staff. Being in charge of the HIV clinic, the clinical officer had substantial experience in conducting performance evaluations and was trained on the CCC evaluation instrument by one of the authors (KWK). A standard evaluation form was used to assess skills in obtaining vital signs, taking histories, using the PDA, making clinical judgments, displaying humanistic qualities, and interacting with clinic staff. Each of these domains was assessed as Superior, Satisfactory, or Unsatisfactory.

The evaluation summary score was obtained by averaging the domain scores and was reported as Superior, Satisfactory or Unsatisfactory. If a domain or an evaluation was identified as unsatisfactory, the in-charge clinical officer either arranged for remediation or if the issue was related to a behaviour (e.g, poor interpersonal interactions, tardiness, or failure to make a scheduled patient visit), discussed the observed conduct and its consequences with the CCC and reinforced project expectations.

After becoming independent, CCCs met with the coordinating clinical officer weekly for the first one to two months to review patient data. Subsequently, when deemed appropriate by the clinical officer, the period between feedback sessions was extended to two weeks and then to every month.

Throughout the study period, the investigators met with the clinical officers and CCCs monthly to discuss barriers and enhancers to the performance of their duties. These meetings allowed CCCs to share their experiences (both successful and unsuccessful) and to aid each other in problem solving and programme development.

### Data sources, management and analysis

Two data sources were used to assess the structure and function of the CCC programme: CCC evaluations; and translated transcripts from the monthly CCC meetings. The proportion of CCCs receiving satisfactory monthly mentoring evaluations and completing clinical training, field mentoring, and one to two years of practice were assessed by summarizing the CCC evaluation forms on an annual basis.

All monthly CCC meetings were audio-taped and were transcribed and translated into English by a trained and experienced research assistant, who was present at all monthly CCC meetings and fluent in Kalenjin, Kiswahili and English. These meetings formed the basis of the continuous feedback and quality improvement programme that was used to assess and perfect the community care system. One of the authors (KWK) performed an impressionistic analysis on the CCC meeting transcripts at regular intervals. Issues identified by this analysis and not previously addressed were investigated as part of the improvement plan. Themes identified and addressed during the content analysis are presented in this paper.

Consecutive patients reporting to clinic who lived in and around Kosirai Division were invited to participate in a cluster-randomized trial (randomization by sub-location) of the CCC intervention. Patients who enrolled in and completed the year-long follow up were asked about their willingness to re-enrol in the programme at the end of the trial. This information was used as a surrogate marker of patient acceptance of, and satisfaction with, the CCC programme.

## Results

### CCC evaluations

Nine CCCs were trained, eight (four men and four women) of whom were assigned to a sub-location and one as a back-up. All eight CCCs previously assigned to a sub-location successfully passed their didactic training and clinical mentoring and entered the field. All of the original CCCs remained in the field for the entire two-year duration of the study. Each CCC managed between eight and 20 patients in their assigned community.

At the end of the first year, 133 formal evaluations had been completed on the eight active CCCs (16 to 17 evaluations per CCC). The CCCs consistently received superior summary scores, with 89% of all scores being superior and the remainder being satisfactory (Table 2). The vast majority of evaluations in each of the assessment areas was rated as superior, with only two evaluations (two different CCCs) being unsatisfactory early in the mentoring period: one in clinical judgment and one in PDA use. Eighty-eight evaluations (11 per CCC) were undertaken in Year 2, again with the vast majority (94%) indicating superior performance by the CCCs.

**Table 2:** CCC evaluations summarized at 1 and 2 years

Study year	Score	Vitals No. (%)	History taking No. (%)	Use of PDA No. (%)	Clinical judgment No. (%)	Humanistic qualities No. (%)	Interaction with staff No. (%)	Summary No. (%)
**1^st ^Year**	*Superior*	109 (90.8)	113 (91.9)	106 (90.6)	103 (83.1)	114 (86.4)	115 (89.1)	118 (88.7)
	
	*Satisfactory*	11 (9.2)	10 (9.1)	10 (8.5)	20 (16.1)	18 (13.6)	14 (10.9)	15 (11.3)
	
	*Unsatisfactory*	0 (0)	0 (0)	1 (0.9)	1 (0.8)	0 (0)	0 (0)	0 (0)

**2^nd ^Year**	*Superior*	88 (100)	87 (98.8)	88 (100)	87 (98.8)	84 (95.5)	81 ((92)	83 (94.3)
	
	*Satisfactory*	0 (0)	1 (1.1)	0 (0)	1 (1.1)	4 (4.5)	6 (6.8)	5 (5.7)
	
	*Unsatisfactory*	0 (0)	0 (0)	0 (0)	0 (0)	0 (0)	1 (1.1)	0 (0)

### Themes arising from monthly meetings

During the first year, six themes emerged from the content analysis of the meeting transcripts: confidentiality and "community" disclosure; roles and responsibilities; logistics; clinical care partnership; ART adherence; and PDA issues. Confidentiality and "community" disclosure were key issues during the first few months after entering the field, when CCCs frequently encountered questions from patients' partners, neighbours and the general population about their activities and role in the community. This experience is exemplified in the following quote from a CCC:

"Her husband followed and as we continued, her husband was waiting for us outside the neighbour's house. When I had finished serving the patient, her husband asked me what we were doing with his wife and I answered him that I was explaining to her about the group based in Mosoriot of which she is a member and I am the leader of that group."

In order to avoid the AIDS label (and its stigma) and ensure patient confidentiality, the CCCs eventually chose to define themselves as health counsellors attached to a project at the rural health centre. To ensure consistent messaging, CCCs requested that clients who were unwilling to disclose their HIV status identify the CCC as a health counsellor to individuals who expressed curiosity. Early in Year 2 of this programme, CCCs recommended that community disclosure and stigma issues be dealt with by an increase in community mobilization activities, as well as by referring patients to support groups. Though stigma remained an issue within the community after 16 months, CCCs no longer reported this as a significant issue for the project.

Two sub-themes emerged within the major theme of CCCs' roles and responsibilities: client expectations and clinic staff expectations. Some clients indicated to the CCCs that they felt that the CCCs should provide them with gifts, such as sugar, or assist in times of financial crisis, as highlighted by this quote by a CCC:

"She [the client] told me to be taking sugar to her during every visit ... She claims that her daughters-in-law rush to her house after my departure because they think that I usually take sugar to her with my large back bag."

Initially, the CCCs felt some discomfort with these requests, but over time, they were able to more clearly define their role as patient care advocates, who could refer patients to social and food services within AMPATH, but who could not provide direct assistance to families. Year 2 meeting transcripts identified no significant conflicts between clients' expectations and responsibilities of the CCCs or the clinic.

The sub-theme of clinic staff expectations emerged during the 10^th ^month of field work. As individuals working from their homes, the CCCs faced issues of competing agendas, and during the 10^th ^month, it was noted that one CCC had failed to make some of his assigned home visits due to his participation in election activities. As a result, his patients were forced to visit the clinic to collect their medications. Other issues encountered during the month included failure of two of the CCCs to come to the clinic for the weekly PDA data downloads and tardiness in getting to monthly meetings. The supervising clinical officer and study staff clearly reinforced the clinical staff's expectations of the CCCs that they adhere to their patient visits, ensure that PDAs are downloaded on a routine basis, and notify the team if they are going to be late for meetings.

During Year 2 in the field, the majority of conflicts with clinic expectations were self-corrected by the CCCs. For example, one CCC failed to acknowledge a vital sign alert at the patient's residence. However, when subsequently reviewing the visit data, the CCC identified the alert and returned to the patient's house for a recheck, which was found to be normal. It was also noted that CCCs were turning off their cellphones during work hours and so could not be reached by the clinic. Clinic expectations about availability were reinforced and this problem did not recur.

With regard to logistics, the CCCs and their clients were given the opportunity to set the times and places for monthly visits. Eventually, most visits occurred at either the patients' or the CCCs' homes because early in the process, CCCs recognized that, due to numerous interruptions and confidentiality concerns, they could not conduct visits at more public venues, such as AMPATH's food distribution site in Mosoriot. As reported by one CCC:

"So, the only thing I saw in the distribution site is that there are so many patients coming to the site and most of them were pleading for help. One of them came to me and asked for help, but I told her to come to the clinic. I also noted that it could be good to meet patients privately to avoid disturbances."

Some patients requested evening visits. However, this was generally discouraged by both the CCCs and the study staff because of safety concerns about travelling after dark. Visit schedules were able to accommodate patients' and CCCs' needs without adding evening visits.

CCCs initially encountered some issues with patients failing to be available at the times and locations scheduled for their monthly visits because of unexpected issues arising, such as funerals, and in rare instances, because the patient had moved without informing the CCC. The strategy developed between the supervising clinical officer and the CCC was to request that patients pass by the homes of their CCCs prior to leaving the area in order to reschedule or postpone appointments. In addition, the clinical officer suggested that the CCCs ask patients about their intention to move at each visit. If CCCs were still unable to contact patients after three separate tries, they were told to refer those patients to AMPATH's outreach team for follow up.

The only logistical issue raised during Year 2 of field work was related to poor cellphone coverage around some of the clients' homes, an issue that could not be directly addressed by the project, but did not prevent the CCCs from performing their duties.

The position of CCCs in the clinical care partnership began evolving within the first month of field work when it became clear that the CCCs were able to identify psychosocial concerns that were not being identified and discussed during clinic visits, such as alcohol abuse, food insecurity, domestic discord, and HIV disclosure issues. Such issues were not always addressed in the clinic, and a referral form was developed that allowed the CCCs to communicate these concerns to the clinical staff.

The importance of the CCCs in the care partnership remained a consistent theme throughout the two years of field work. In addition to identifying psychosocial issues, CCCs provided trusted and reliable linkages between AMPATH's pharmacy, outreach and clinical teams and the patients to deal with important issues, such as adherence to medications or clinic appointments.

Over the two years of field work, the theme of cART adherence repeatedly emerged during the monthly meetings. The initial adherence issue was what to do with the excess tablets identified during monthly pill counts. Because of the complexity of collecting and returning excess pills, it was decided that the CCCs should simply record the number of excess tablets and allow the clinic to reconcile the patients' medications. The CCCs felt that they were more accurate at assessing adherence than the clinic because patients could not hide their pills during home visits. Thus, as one CCC put it:

"I learnt that patients never cheat when they are at their homes than when they come here at the clinic because most of them can give you the pills to count, but they sometimes leave other pills at home when coming to the clinic."

In addition to monitoring adherence, CCCs were involved in adherence support, which included identifying issues that adversely impacted medication adherence (e.g., religious beliefs, alcohol use and domestic issues), explaining changes in the number of pills that needed to be taken (e.g., when DDI 200 mg tablets were out of stock, they had to be replaced with four 50 mg tablets), and explaining changes in formulation (e.g., when combivir replaced individual zidovudine and lamivudine). One example of information that the CCCs were able to glean about adherence beliefs is as follows:

"... both clients had the same problems of not adhering to their drugs because of their religious faith ... The patient had relied most on church norms and wanted to leave the drugs. So, we told him that going to church was not bad and trusting in the Lord was good, but he should do both."

One CCC accompanied her poorly adherent patient to the clinic in order to provide support to the clinic staff in reinforcing adherence behaviours. CCCs also played a key role in tracking patients who had been displaced during the post-election violence that occurred during January and February 2008.

CCCs initially had some difficulties with using the PDAs in the field. There were problems keeping the PDAs' batteries charged, as well as issues with data entry. Paper forms were distributed to all CCCs to be used for back up when their PDAs lost charge or the CCCs had difficulties with data entry. A PDA refresher course was given four months into field work, and a tutor was assigned to the two CCCs who were having the most difficultly with data entry. PDA issues were cited much less frequently as problems during Year 2, when the most significant problems encountered were: a stolen PDA, which was subsequently recovered, but was not functional upon retrieval; and a problem with the study computer preventing the timely download of data from the PDAs for approximately a month.

The only new theme that emerged during the second year of the project was the unexpectedly large number of pregnancies among stable patients being cared for by the CCCs. The CCCs felt that the majority of these pregnancies were unintended, and there was general discussion of how to better serve the reproductive health needs of their clients. However, other than general recommendations, such as referring patients to family planning services, there was no significant resolution of this issue.

### Patient acceptance

The CCCs described patient acceptance of their role early during their field work, as outlined in this quote:

"... some of the patients are very happy to get their drugs at their homes. It is good now because during our visits, we discuss many confidential issues that we cannot reveal to anyone what we have discussed."

By December 2006, CCCs reported encountering patients in the field who told them that they wanted to enrol in the CCC programme. By the end of the trial, of those not lost to programme, 64% (56 of 87) in the intervention arm and 52% (58 of 103) in the control arm were willing to continue in the programme (p = 0.26). Individuals were not asked why they chose not to re-enrol. However, study staff felt that study visit fatigue was a factor.

## Discussion

The CCC model of task shifting, outlined in this paper, allowed us to operationalize Recommendation 20 of WHO's Task Shifting, global recommendations and guidelines, which states: "Community health workers, including people living with HIV/AIDS, can safely and effectively provide specific HIV services both in a health facility and in the community in the context of service delivery according to the task shifting approach"[15].

Our CCC model was found to be acceptable to the clinic staff, the patients and the CCCs themselves, it was feasible, and it accomplished an approximately 50% reduction in clinic visits by the intervention group. Like the *accompagnateurs *in the Haitian model of care,

CCCs were found to enhance the care team by providing sometimes unexpected insights into patient adherence and psychosocial issues impacting care [18]. In addition, they played a key role in facilitating communication between the clinic and the patient: the patients saw the CCCs as advocates, while the clinic considered them to be an extension of the clinic staff.

Monitoring and evaluation has been a significant concern among those advocating task shifting as a means of improving access to ART [15]. Our continuous feedback and quality improvement model allowed for uninterrupted monitoring and evaluation of the programme and facilitated rapid changes in the programme to improve functioning. Though minor issues in job performance of the CCCs were noted, the monthly CCC meeting allowed for problems to be addressed and corrected in a timely fashion. Use of PDAs allowed for the clinical officer in charge of the Mosoriot HIV clinic to assess the performance of home visits, as well as to provide consistent evaluation and referral of patients to the clinic. The project is currently assessing patient level data to determine the impact of the CCC on adherence, clinical outcomes (viral load and CD4 cell count) and patient perception of stigma.

We have learned four major lessons from this project. The first is that despite our provision of HIV treatment in the Kosirai Division since 2001, HIV disclosure remains an issue for our patients. As such, we recommend that programmes in our region that provide community-based HIV care consider how to represent and package this care in a way that avoids the AIDS label, much as the CCCs did by defining themselves as health counsellors.

Second, we found that it took longer than anticipated for the CCCs to adapt to new technologies, particularly the use of PDAs. In future, we would recommend a full week being devoted to PDA didactics and structured exercises to ensure competency prior to field entry. However, our experience shows that new technologies, such as PDAs, cellphones, and electronic scales, thermometers and pulse oximeters, can overcome otherwise overwhelming logistical barriers to high-quality continuous care. The barriers include the lack of paved roads, especially during the rainy seasons, and the cost of public transportation.

The third lesson is that patient referral must function bi-directionally and that mechanisms should be put in place to facilitate CCC referral to the clinic and clinic referral of follow up of particular issues to the CCCs.

The fourth lesson is that such programmes are not without cost. There are the costs of training and mentoring CCCs (which in our case, were absorbed by the existing clinical programme), CCC salaries, equipment (including PDAs), PDA maintenance, and for patients in far-flung areas, the cost of transportation. However, since the goal of the CCC model is to reduce visits to the health centre, some or all of these costs should be offset by reducing health centre personnel time needed to care for CCC patients. In addition, for other programmes considering providing similar services, it is impossible to overstress the importance of identifying fully committed and engaged individuals to be CCCs.

## Conclusion

In conclusion, we found that an ART delivery model that shifted patient monitoring and ART dispensing tasks into the community by HIV-infected patients was both acceptable and feasible. Integrating this cadre to the care team enhanced the team's understanding of the psychosocial issues that impact on an individual patient's care. These findings provide advocacy, and support further exploration of the role of HIV-infected lay individuals in providing specific HIV-care services.

## Competing interests

The authors declare that they have no competing interests.

## Authors' contributions

KKW conceptualized and designed the study, developed the data collection instruments, performed the primary data analysis, had primary responsibility for interpretation of the data, and drafted the manuscript. JES assisted in the conceptualization and design of the study, development of the data collection instruments, interpretation of the results, and provided final approval to the manuscript. HMS assisted in data analysis, interpretation of results, and provided final approval of the manuscript. RV assisted in data collection, data analysis, interpretation of results, and contributed to the drafting of the manuscript. EKK performed data collection, interpretation of the results, and provided final approval of the manuscript. VTJ performed data collection, interpretation of the results, and provided final approval of the manuscript. LJB performed data collection, interpretation of the results, and provided final approval of the manuscript. AEC designed the PDA programme and provided final approval of the manuscript. WMT assisted in conceptualization and design of the study, interpretation of the results, and contributed to the drafting of the manuscript. SK assisted in conceptualization and design of the study, interpretation of the results, and approved the final manuscript. All authors have read and approved the final manuscript.
